# Beyond ‘charting outcomes’ in the radiation oncology match: analysis of self-reported applicant data

**DOI:** 10.1080/10872981.2018.1489691

**Published:** 2018-06-26

**Authors:** Samuel Jang, Stephen A Rosenberg, Craig Hullett, Kristin A Bradley, Randall J Kimple

**Affiliations:** a Department of Human Oncology, University of Wisconsin School of Medicine and Public Health, Madison, WI, USA; b Department of Radiation Oncology, H. Lee Moffitt Cancer Center and Research Institute, Tampa, FL, USA; c University of Wisconsin Carbone Comprehensive Cancer Center, University of Wisconsin School of Medicine and Public Health, Madison, WI, USA

**Keywords:** Radiation oncology, applicant, match, online database, student doctor network

## Abstract

The Charting Outcomes resource is useful in gauging an applicant’s competiveness for a given specialty. However, many variables are not reported in Charting Outcomes that may influence an applicant’s ability to match. A significant proportion of applicants record their experiences in an anonymous, self-reported applicant spreadsheet. We analyzed factors associated with a successful match using this dataset to test the hypothesis that research productivity and high academic performance correlates with success rates. A retrospective analysis of “RadOnc Interview Spreadsheet” for the 2015, 2016, and 2017 radiation oncology match was performed. Data were accessed via studentdoctor.net. Board scores, research characteristics, Sub-I participation, and interview invitation rates were available. Mann-Whitney U, Kruskal-Wallis, and chi-square tests were used for statistical analysis. When possible, results were compared to those reported in the National Residency Match Program’s “Charting Outcomes” report. A total of 158 applicants were examined for the applicant characteristics. Applicants applied to a median of 61 programs and received a median of 14 interviews. The mean step 1 score was 248 (range: 198 to 272) and most were in the highest grade point average quartile (68.3%). 21.7% participated in additional research year(s), and 19% obtained a PhD. The majority of applicants took three radiation oncology electives (48.7%). On multivariate analysis, alpha-omega-alpha (AOA) honors society status (p=0.033), participating in a research year (p=0.001) and number of journal publications (p=0.047) significantly correlated with higher interview invitation rates. In summary, this study identifies important considerations for radiation oncology applicants that have not been previously reported, such as induction into AOA and number of journal publications.

## Introduction

Radiation oncology is a competitive specialty in which approximately 200 medical students per year apply to residency programs [1]. The number of applicants has been steadily rising in the past decade [2]. The National Resident Match Program (NRMP) publishes ‘Charting Outcomes in the Match’ which summarizes data from residency and match applications [3,4]. However, the data collected and released by the NRMP remains limited; for example, research activity combines the number of publications and presentations, and does not separately identify first author publications and data on elective rotations is not reported.

For the past three residency application seasons (2014–2017), senior medical students aspiring to match into a radiation oncology program have been inputting their own statistics and characteristics into an open-access database publicized in Student Doctor Network (SDN), an online forum focused for medical students. Herein, we summarize the information in the self-reported database to provide additional information not available through the NRMP publications.

## Methods and materials

### Database

‘RadOnc Interview Spreadsheet,’ an anonymous, prospectively self-reported data from applicants applying to radiation oncology programs, was analyzed for the 2015, 2016, and 2017 match. This database is available in Student Doctor Network (SDN; https://forums.studentdoctor.net/threads/2016-2017-radonc-interview-spreadsheet.1222027/), which we accessed on 10 July 2017 for analysis. Our Institutional Review Board deemed this study not-human subjects research. During the interview season, a combined total of 162 students reported their personal and application characteristics. International applicants (*n* = 1) and nonsensical data (*n* = 3) (e.g., step 1 score with decimals) were excluded for a total of 158 applicants (53, 56, and 49 in 2015, 2016, and 2017, respectively). Given that there were 592 total applicants during these cycles according to NRMP data [1,5,6], 27% (158/592) of the total applicant pool were analyzed in this study.

### Statistical analysis

The data were analyzed with Excel (Microsoft, Redmond, WA, USA) and SPSS statistical software 23 (IBM Corp, Armonk, NY, USA). Mann-Whitney U tests and Kruskal-Wallis non-parametric tests were used to compare continuous variables. SPSS medians test compared medians of independent samples. Categorical variables were analyzed with Chi-squared test. All variations are reported in standard deviation. *P* value < 0.05 was considered significant.

## Results

### Applicant characteristics

Data was available for 158 U.S. radiation oncology resident applicants. Characteristics of the variables reported in Radiation Oncology Interview Spreadsheet are summarized in Table 1. Applicants applied to a median of 61 programs (range: 17–89) and received a median of 14 interviews (range: 0–47). The USMLE Step scores were reported for over half the population (64%). Mean step 1 and step 2 scores were 248 (range: 198–272) and 256 (range: 216–278), respectively. Only one applicant of three who scored below 200 on the step 1 exam disclosed the number of interviews received and conveyed receiving no interviews after applying to 79 programs. A considerable number of applicants were in the highest GPA quartile in their respective institutions (68%) and were inducted into the alpha omega alpha (AOA) society (38%). The most common number of radiation oncology elective rotations (including one in their home institution) completed was 3 (range: 0–5).

**Table 1.** Applicant academic and interview season characteristics.

Step 1 score (*n* = 158)		# of RO rotations (*n* = 156)	
Median	250	0 (%)	2 (1.3)
Mean (SD)	248.4 (14.3)	1 (%)	10 (6.4)
Step 2 submitted (*n* = 156)		2 (%)	37 (23.7)
No (%)	57 (36.5)	3 (%)	76 (48.7)
Yes (%)	98 (63.5)	4 (%)	30 (19.2)
Step 2 score, if submitted (*n* = 155)		5 (%)	1 (0.6)
Median	258	Programs applied (*n* = 151)	
Mean	255.9 (13.2)	Median	61
GPA quartile (*n* = 123)		Mean (SD)	63.6 (17.6)
1^st^ (%)	84 (68.3)	Interviews received (*n* = 129)	
2^nd^ (%)	24 (19.5)	Median	14
3^rd^ (%)	12 (9.8)	Mean (SD)	15.7 (8.7)
4^th^ (%)	3 (2.4)	Couples match (*n* = 151)	
AOA status (*n* = 146)		No (%)	146 (96.7)
No (%)	90 (61.6)	Yes (%)	5 (3.3)
Yes (%)	56 (38.4)	Match on rank list (*n* = 85)	
Top 40 NIH funded school^a^ (*n* = 42)		1 (%)	38 (44.7)
No (%)	26 (61.9)	2 (%)	17 (20.0)
Yes (%)	16 (38.1)	3 (%)	9 (10.6)
PhD (*n* = 153)		4 (%)	6 (7.1)
No (%)	124 (81.0)	5 (%)	5 (5.9)
Yes (%)	29 (19.0)	6 (%)	7 (8.2)
Research year (*n* = 152)		7 (%)	0 (0.0)
No (%)	119 (78.3)	8 (%)	1 (1.2)
Yes (%)	33 (21.7)	Did not match (%)	2 (2.4)

GPA: grade point average; NIH: National Institute of Health; AOA: Alpha Omega Alpha; RO: radiation oncology ^a^Not reported on 2015 and 2016 match applicant spreadsheets

Only 85 applicants reported where in their personal rank list they matched, including two who did not match. Of those who reported, the majority matched at their top choice (45%). The five applicants who couples matched scored 242–260 on step 1, were all in the first GPA quartile, participated in 2–3 elective rotations and had variable participation in research (3–22 publications, presentations, and posters listed on ERAS). Four of them were AOA members. They ultimately received 11–47 interviews and matched first – eighth on their rank list.

### Research productivity

Applicants reported whether they participated in an additional research year(s) (RY) or were graduating with a PhD. The total number of publications, presentations, and posters reported on Electronic Residency Application Service (ERAS) ranged from 0 to 50. The number of journal publications and first author publication ranged from 0 to 20 and 0 to 9, respectively. The two applicants who reported no research productivity and eight applicants who reported no journal publications all matched. The research productivity of students who did and did not participate in these additional research experiences outside of their core curriculum are summarized in Table 2. Expectedly, PhD students reported the most research productivity followed by RY students. Students without additional research experience had a median of three journal publications and one first author journal publication; however it was not reported whether these were produced during or before medical school. Interestingly, RY students had more radiation oncology specific publications, presentations, and posters reported on ERAS compared to PhD students (*P* = 0.009).

**Table 2.** Research productivity by research experience.

		No Gap year (*n* = 92)	Research year (*n* = 31)	PhD (*n* = 29)	*P*-value
# of publications, presentations, and posters on ERAS	Median Mean (SD)	9.0 10.9 (8.4)	13.0 14.1 (7.5)	18.0 18.1 (8.2)	< 0.001 < 0.001
# of first author publications, presentations and posters on ERAS^a^	Median Mean (SD)	4.0 5.6 (4.7)	8.5 8.5 (5.8)	10.0 11.5 (6.2)	0.001 0.001
# of rad onc specific publications, presentations, and posters on ERAS	Median Mean (SD)	3.5 5.8 (8.1)	8.5 8.2 (7.3)	2.0 6.0 (9.2)	0.029 0.009
# of journal publications	Median Mean (SD)	3.0 3.5 (3.5)	4.5 4.6 (2.9)	8.5 8.9 (4.0)	< 0.001 < 0.001
# of first author journal publications^a^	Median Mean (SD)	1.0 1.4 (1.5)	2.0 2.6 (2.3)	4.0 4.2 (2.0)	0.002 < 0.001

ERAS: Electronic Residency Application Services; rad onc: radiation oncology ^a^Not reported on 2015 match applicant spreadsheet

### Interview invite rate

The percentage of interviews received was calculated from the numbers of applications submitted and the number of interview invitations received. The number of elective rotations taken, number of radiation oncology specific research activities, decision to submit a step 2 score, and attending a top 40 NIH funded institution did not significantly correlate with interview invitations on univariate analysis (supplementary Table S1 and S2). On multivariable regression analysis, AOA status, taking a RY (but not PhD), and number of journal publications remained significant (Table 3). To avoid multicollinearity, only the number of journal publications was chosen to be included in the multivariate analysis to represent the most meaningful research productivity. With backward eliminated multivariable regression analysis, no significant changes were seen in the parameters associated with higher interview rates.

**Table 3.** Multivariable regression analysis of factor associated with higher interview rates.

	Standardized beta coefficient	*P*-value
Step 1 (*n* = 126)	0.025	0.822
Step 2, if reported (*n* = 83)	0.177	0.139
AOA status (*n* = 122)	0.241	0.033
Research year (*n* = 127)	0.361	0.001
PhD (*n* = 127)	0.020	0.867
# journal publications (*n* = 126)	0.240	0.047

AOA: Alpha Omega Alpha

### Data validation

To address concerns regarding data quality, we attempted to validate the data in these online datasets. We emailed a random cohort of radiation oncology residents asking them to confirm the validity of their anonymously reported data by revisiting the spreadsheet and emailing the authors. Unfortunately, of 94 residents attempted to be contacted who may or may not have contributed to the dataset, only nine responded to our request. Of these, all verified that their data was correct. We also compared the applicant characteristics of the online dataset to those of matched applicants in ‘Charting Outcomes in the Match 2016,’ (Supplementary Table S3). Similar step 1 and step 2 scores were reported. There were more AOA members and fewer PhDs in the self-reported data. The number of publications, number of programs ranked, and percent graduating from top 40 medical schools showed slight differences. There is no ‘Charting Outcomes in the Match’ published for the 2015 and the 2017 match.

## Discussion

Radiation oncology is currently among the most competitive residency choices for medical school graduates. Prospective residents use multiple tools to gauge their competitiveness and improve their chances of a successful match process [1,3–8]. Today’s radiation oncology applicants have high national board scores, are academically talented, and engage in significant research experiences [3,4]. These findings were confirmed within the self-reported dataset analyzed here. Additional characteristics reported in our study that have not previously been described include the percent of applicants reporting their Step 2 score, their GPA quartile, the number of radiation oncology elective rotations completed, the number of applications submitted, the number of interviews received, the number of applicants participating in the couples match, and where in their personal rank list applicants matched.

It is well-known that radiation oncology programs generally value research experience. We analyzed in detail the publication productivity of students with PhD degree, students who participated in a RY(s), and students without additional research activity. Expectedly, the students with commitment to additional research experiences had higher publication numbers, which correlated with higher interview invitation rates. In the database analyzed, students who took a RY reported higher productivity in radiation oncology specific publications compared to PhD students since PhD students may have pursued a thesis in another field of study. It is also possible that PhD students pursue more basic science projects that typically take longer to complete, leading to a lower number of publications.

There are clear limitations to this study that we have attempted to minimize through our approach. Applicants could have inputted incorrect information into the anonymous self-reported online database. As the data is meant to be used to help fellow and future applicants, we believe that there is little reason to abuse the dataset. We attempted to validate the integrity of the SDN data by emailing current applicants to confirm the accuracy of their data, but received few responses. Another limitation is that the characteristics of the applicants who choose to self-report their stats on the SDN database, even if anonymous, may be different from those who do not access SDN or choose not to post their stats. Given the nature of self-reported anonymous data, students and mentors should interpret our data with careful circumspection. Despite these limitations, our data seems to closely reflect the applicant pool reported in the recently published ‘Charting Outcomes in the Match 2016.’ The minor exceptions were the higher percentage of AOA members and lower percentage of applicants with PhD in the online dataset compared to the NRMP data. These small differences between the SDN and NRMP data were also highlighted in a recent publication and were similar to our study [9]. Finally, we are unable to analyze personality traits and individual characteristics that can be very important in finalizing program rank lists. These qualitative characteristics are most likely evaluated by letter of recommendations, personal statements, and interactions during elective rotations and interviews.

## Conclusion

This study summarizes the self-reported applicant database popularized by the SDN website over three match cycles. This analysis identifies factors associated with higher rates of interview invitations to radiation oncology residency programs. Our analysis found that higher research productivity, especially participation in additional research experiences and number of journal publications, translates into higher number of interview invitations.
