# Supplementary material for: Beyond ‘charting outcomes’ in the radiation oncology match: analysis of self-reported applicant data
Source: Med Educ Online. 2018 Jun 26;23(1):1489691. doi: 10.1080/10872981.2018.1489691 (PMC6022246; doi:10.1080/10872981.2018.1489691)
Supplement: Supplemental Material [file ZMEO_A_1489691_SM6978.docx]

**Supplementary Table S1. Categorical factors associated with higher rate of interview invitations – univariate analysis**

|  | # of applications | | | |  | % interview received | | | |
| --- | --- | --- | --- | --- | --- | --- | --- | --- | --- |
|  | N | Median | Mean (SD) | P-value |  | N | Median | Mean (SD) | P-value |
| AOA |  |  |  |  |  |  |  |  |  |
| No | 88 | 68.5 | 65.2 (18.4) | 0.295 |  | 76 | 18.9 | 21.7 (12.5) | <0.001 |
| Yes | 55 | 60 | 62.1 (15.3) |  |  | 46 | 30.0 | 32.7 (16.9) |  |
| Research |  |  |  |  |  |  |  |  |  |
| No gap | 89 | 70 | 66.8 (16.9) | 0.322 |  | 69 | 20.7 | 22.7 (13.3) | 0.013 |
| Research year | 31 | 64 | 63.4 (19.2) |  |  | 29 | 28.0 | 33.0 (19.6) |  |
| PhD | 29 | 50 | 52.4 (13.2) |  |  | 29 | 28.6 | 29.7 (13.5) |  |
| # of RO rotations |  |  |  |  |  |  |  |  |  |
| 1 | 10 | 50.5 | 51.4 (18.2) | <0.001 |  | 10 | 34.4 | 36.5 (16.2) | 0.202 |
| 2 | 35 | 60 | 62.5 (17.2) |  |  | 29 | 23.0 | 25.7 (15.7) |  |
| 3 | 72 | 62.5 | 64.3 (16.0) |  |  | 63 | 21.3 | 25.7 (14.7) |  |
| 4 | 29 | 71 | 65.9 (20.0) |  |  | 23 | 27.5 | 27.0 (16.1) |  |
| Step 2 submitted |  |  |  |  |  |  |  |  |  |
| No | 57 | 60 | 62.4 (18.8) | 0.771 |  | 44 | 26.4 | 27.2 (15.0) | 0.875 |
| Yes | 92 | 64 | 63.8 (16.7) |  |  | 83 | 21.4 | 26.3 (16.0) |  |
| Top 40 NIH funded school |  |  |  |  |  |  |  |  |  |
| No | 88 | 68.5 | 62.9 (20.6) | 0.322 |  | 24 | 20.2 | 23.4 (15.1) | 0.885 |
| Yes | 55 | 60 | 66.9 (14.5) |  |  | 15 | 19.5 | 21.4 (11.8) |  |

AOA = Alpha Omega Alpha; RO = radiation oncology; NIH = National Institute of Health

**Supplementary Table S2. Nominal factors associated with higher rate of interview invitations – univariate analysis**

|  | N | Pearson correlation | B | P-value |
| --- | --- | --- | --- | --- |
| Step 1 score | 126 | 0.189 | 0.245 | 0.034 |
| Step 2 score, if submitted | 83 | 0.286 | 0.353 | 0.009 |
| # of publications, presentations and posters reported on ERAS | 127 | 0.257 | 0.474 | 0.003 |
| **#** of 1^st^ author publications, presentations, and posters reported on ERAS | 68 | 0.322 | 0.928 | 0.007 |
| # of rad onc specific publications, presentations, and posters reported on ERAS | 125 | 0.14 | 0.327 | 0.117 |
| **#** of journal publications | 126 | 0.33 | 1.175 | <0.001 |
| # of 1^st^ author journal publications | 69 | 0.314 | 2.319 | 0.009 |

ERAS= Electronic Residency Application Services; Rad onc = radiation oncology

**Supplementary Table S3. Comparison of Student Doctor Network data to Charting Outcomes in the Match data**

|  | Self-reported online dataset | Charting Outcomes 2016, matched applicants |
| --- | --- | --- |
| Step 1 score (mean) | 248 | 247 |
| Step 2 score (mean) | 256 | 251 |
| # of abstracts, presentations, and publications (mean) | 11.6 | 12.7 |
| # of contiguous ranks (mean) | 12.2 | 11.6 |
| % who are AOA members | 38.4% | 27.5% |
| % who have a PhD | 19.0% | 24.8% |
| % who graduated from one of the 40 U.S. medical schools with the highest NIH funding | 38.1% | 41.6% |

AOA = Alpha Omega Alpha; NIH = National Institute of Health
